# Effect of Semisolid and Heat Treatment Process on Microstructural Refinement of Al–7Si Alloy

**DOI:** 10.3390/ma16031086

**Published:** 2023-01-27

**Authors:** Mohamed Abdelgawad Gebril, Mohd Zaidi Omar, Intan Fadhlina Mohamed, Norinsan Kamil Othman, Dawod Mohamed Elabar, Farag Ibrahim Haider, Saziana Samat, Osama M. Irfan

**Affiliations:** 1 Department of Mechanical Engineering, Faculty of Engineering, University of Benghazi, Benghazi 16063, Libya; 2 Department of Mechanical and Manufacturing Engineering, Faculty of Engineering and Built Environment, Universiti Kebangsaan Malaysia, UKM, Bangi 43600, Malaysia; 3 Department of Applied Physics, Faculty of Science and Technology, Universiti Kebangsaan Malaysia, UKM, Bangi 43600, Malaysia; 4 Department of Mechanical Engineering, College of Engineering, Qassim University, Buraydah 51452, Saudi Arabia; 5 Mechanical Department, Beni Suef University, Beni Suef 62746, Egypt

**Keywords:** Al–Si alloy, cooling slope, thixoformed, heat treatment, corrosion resistance

## Abstract

Improving the engineering properties of Al–7Si cast alloys (300 series) provides an attractive alternative to automotive and aircraft engine industries. The solubility limit of silicon (Si) in Al contributes to the precipitation of flake-shaped Si particles with sharp edges, which function as a stress riser and promote crack propagation during the eutectic phase while also weakening the protective layer’s durability. In this study, the impact of microstructure refinement of Al–7Si alloys by using cooling slope, thixoforming and the T6 heat treatment process on hardness and corrosion resistance behavior was investigated. Results showed that the microstructures of the as-cast alloy had a very coarse dendritic shape, whereas the dendritic transferred to the globular α-Al phase, and the Si particles were replaced into a lamellar- or acicular-like shape after the cooling slope and thixoforming process, respectively. The as-cast, cooling slope and thixoformed samples were subjected to the T6 heat treatment process, which enhanced the hardness to 79, 99 and 104 HV, respectively, due to Si particle refinement. The potentiodynamic test revealed that the corrosion rate dropped to 0.00790 and 0.00736 mmpy^−1^ in the heat-treated cooling slope and thixoforming samples. This finding can be attributed to the substantially refined Si particles and reduced eutectic phase area due to the smaller cathodic to anodic area ratio.

## 1. Introduction

Al–7Si alloys can be used to cast complex geometry components in aircraft and automatic industries because of their good corrosion resistance, castability and weldability [1,2]. However, using plate-like Si is not as desirable in achieving better mechanical properties [3]. Reports showed that its strength and ductility cannot cope with the ever-increasing demands of some critical structural components [4]. During deformation, the coarse acicular Si particles and their sharp edges might cause early fractures. As a result, the room temperature’s workability, which is mostly determined by ductility values, is weakened [5,6,7]. Therefore, the as-cast Al–Si alloys should be able to alter the morphology of the eutectic Si from flake-like to a circular or spherical shape. Consequently, different modification approaches that can be used for the structure modification of Si in unmodified Al–Si alloys have been proposed to enhance their electrochemical and mechanical properties. In order to enhance the microstructural morphology of Al–Si alloys with dendritic α-Al phase and coarse Si particles, several methods can be used to satisfy industry requirements in several sectors. The utilisation of semi-solid metal (SSM) enhanced the engineering properties through the microstructural change from coarse dendritic α-Al grains to a globular shape as well as the change from flaky Si particles to an acicular shape. Furthermore, these techniques are expected to serve as alternatives to traditional casting processes because they offer the possibility of constructing defect-free, heat treatable and near-net shape components at low cost [8,9]. This approach produces materials that possess homogeneous globular microstructures that are characterised by decreased segregation and porosity [10,11].

The SSM technique has the potential to produce materials in the semisolid region at temperatures exceeding the solidus but lower than the liquidus temperature, where the solid and liquid phases coexist to create a near-net shape product [12]. Two of the most well-known developed methods for producing semi-solid components are thixoforming and cooling slope. These processes have been reported to have the advantages of producing complex products, reducing porosity and improving alloy properties. In the cooling slope process, the alloy is continuously agitated and maintained in the semisolid state before it is poured and injected into the die. The thixocasting process involves partially remelting the billet in the semisolid region [13]. Several techniques have been introduced for producing the desired suitable nondendritic microstructure. Amongst all techniques, the cooling slope (C.S) casting process is considered to be the simplest method of semi-solid metal casting; it requires minimal equipment and operational costs [14,15,16,17]. The technique promotes homogeneous globular microstructures that reduce segregation and porosity [10,18]. SSM has the ability to improve the mechanical properties [17,19,20] and corrosion resistance [10,21] of Al alloys.

Another technique, which can be employed to enhance mechanical and electro-chemical properties, is heat treatment. As a result of heat treatment, the workability and mechanical and electrochemical characteristics of the Al–Si alloy are improved as the lamellar or acicular Si is changed to a globular form, increasing the alloy ductility. The first benefit is achieved through spheroidisation of eutectic Si in the microstructure. The Si particles are transformed from an angular to a spherical shape through the break down and coarsening process. The second precipitation of Mg_2_Si after the aging process enhances the mechanical properties. This transformation of the Si morphology of the semisolid alloy after being subjected to heat treatment (heat-treated semisolid samples) into a spherical shape results in better ductility and higher fatigue strength of the semisolid casting alloy [22,23,24] as well as improved corrosion resistance [25,26]. The large eutectic Si particles in the as-cast alloy retain their lamellar-like shape in the final microstructure in semisolid casting after the heat treatment process [27]. Even though the morphology of Si has transformed into an angular spherical-like shape, the presence of a relatively large area of the eutectic phase containing Si particles with lamellar- or acicular-like shape promotes crack propagation through the eutectic phase and reduces corrosion resistance due to the formation of a large potentially susceptible corroded area. When corrosion occurs, the corrosion product is stacked on the surface, promoting the formation of the occluded corrosion cell, thereby exacerbating the corrosion damage [10,28]. This study investigated the effects of microstructural change via SSM and T6 heat treatment on hardness and corrosion resistance. The tests were carried out under the following processing conditions to compare the results: (a) individual refining method—cooling slope, thixoforming and heat treatment; (b) combined semisolid and T6 heat treatment.

## 2. Materials and Methods

In this study, the cast commercial Al–7Si alloy in ingot with an initial dimension of 70 × 40 × 130 mm was used. The as-cast material of the cooling slope casting process was melted in a graphite crucible at 750 °C. For this process, the cooling slope used was stainless steel with tilt angle of 60° and slope length of 400 mm. This study selected a pouring temperature of 620 °C to limit the melt overheating. The melt was poured into a vertical mould from a stainless-steel slope, and then the mold and the contents were quenched in water. Samples were sectioned to dimensions of 30 X 120 mm for thixoforming, and the grains were spheriodized by rapidly reheating to 585 °C for 5 min. A hydraulic cylinder (Vistech Technology Sdn Bhd, Selangor, Malaysia) press with a load of 20 kN and a maximum compression velocity of 85 mm/s was used to compress the samples. The thixoforming unit’s die was preheated to 300 °C. A calibrated K-type thermocouple (RS Components Sdn Bhd, KualaLumpur, Malaysia) was placed 8 mm from the top of the slug to monitor the heating. Argon gas flowed at 2.5 l/min and was capped with a stainless-steel cover to avoid oxidation during the thixoforming process. According to the T6 technique, the as-cast, cooling slope-cast and thixoformed samples were subjected to a heat treatment process that included an eight-hour sequence of solution treatment at 535 °C, water quenching at 60 °C and a three-hour aging process at 180 °C [29].

The samples were subsequently examined under an optical microscope (OM, Olympus Corporation, Tokyo, Japan) and field emission scanning electron microscope (FESEM, Zeiss, Oberkochen, Germany). In addition, for elemental analysis, an energy dispersive x-ray (EDX) (fitted to FESEM) was used. The hardness of these materials was measured using a Vickers hardness tester (micro-Vickers hardness tester Zwick, Zwickau, Germany; ZHV) with an applied load of 100 g and a dwell time of 15 s. These samples were also processed for microstructure investigation using Si carbide (SiC) papers with grits ranging from 180 to 2000, and then polished with 1 and 3 µm diamond paste (Al_2_O_3_). Meanwhile, Keller’s reagent (1% HF, 1.5% HCl, 2.5% HNO_3_, H_2_O solution) was used as an etchant for the etching operation. The grain size was measured using quantitative metallography analysis in accordance with the ASTM E112 standard. The dimension of Si particles (width and length) was determined using the Smart Tiffv2 program, with at least 200 particles in each sample. Then, the electrochemical experiment was carried out at room temperature with a pH of 6.5 in a naturally aerated 3.5 percent NaCl solution. The corrosion rate of these samples was measured using the potentiostat GAMRY 3.2 (Aseptec Sdn Bhd, Selangor, Malaysia). A three-electrode cell with test material (as the working electrode), graphite (as the counter electrode) and silver or silver chloride ((Ag or AgCl) as the reference electrode) was used. Each sample was essentially placed in epoxy that had been ventilated for 24 h. Prior to each corrosion test, these samples were ground to a fineness of up to 1200-grit SiC.

## 3. Results

### 3.1. Microstructure of as-cast Al–Si Alloy

**Figure 1:**
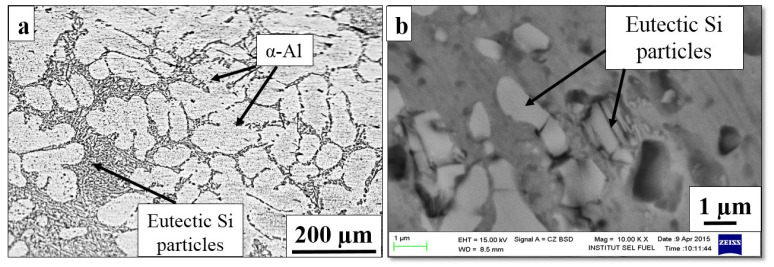
Microstructural features of as-cast Al–7Si alloy: (**a**) optical micrograph; (**b**) SEM morphology of eutectic Si.

Figure 1a shows the optical micrographs of the as-cast sample revealing the typical microstructure of the unmodified hypoeutectic Al–7Si alloy.

The primary α-Al phase, which is surrounded by coarse flaky Si particles in the eutectic phase (dark phase), is formed during the initial solidification phase. The dendritic grain size is approximately 180 µm with a coarse eutectic mixture phase. Figure 1b shows the SEM morphology of a flake-like shape Si particle with sharp edges with an average size of 4.56 ± 1.18 μm.

### 3.2. Microstructure after Cooling Slope Process

**Figure 2:**
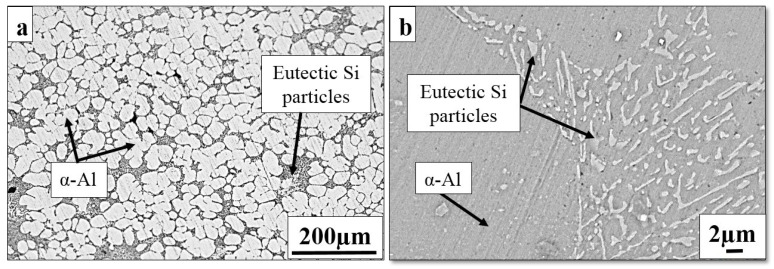
Microstructural features of the cooling slope Al–7Si alloy by (**a**) optical micrograph and (**b**) SEM morphology of eutectic Si.

Figure 2a shows the optical micrographs after cooling slope casting over a cooling slope. The primary α-Al dendrites in the as-cast alloy were completely replaced with an almost globular shape, which was surrounded with the coarse eutectic phase. The α-Al grains transformed into a spherical shape with an average size of 54 µm. The temperature of the molten metal dissipated when poured over the cooling slope, followed by a sudden temperature drop below the liquidus temperature. Partial coagulation of the α-Al phase can occur on the cooling slope. The nucleated α-Al was detached due to shear action and trapped in the flowing melt; the moulds were collected before they grew into a dendritic shape, as also discussed by Birol [30]. As illustrated in Figure 2b, the cooling slope exhibited a change in the eutectic Si morphology into a combination of lamellar and relatively round shapes with a 3.2 ± 0.57 µm average size, with the involvement of the cooling slope. The α-Al microstructure surrounded by the eutectic phase in the cooling slope sample displayed a higher level of uniformity than the as-cast alloy. An earlier study revealed that the cooling slope casting of the alloy obtained refined Si particles [31].

### 3.3. Microstructure after Thixoforming Process

**Figure 3:**
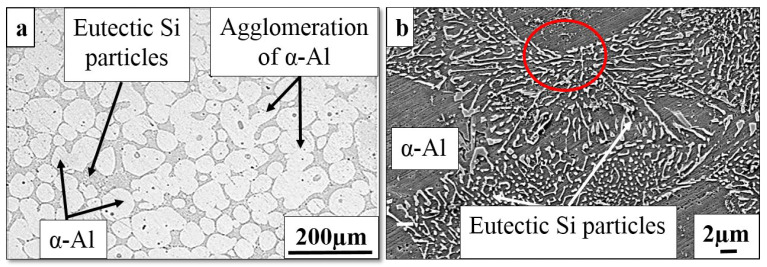
Microstructural features of thixoformed Al–7Si alloy by (**a**) optical micrograph and (**b**) SEM morphology of eutectic Si.

The feed material formed by the cooling slope was subjected to a reheating process, which occurred in a semisolid condition. At the temperature level for the reheating process, the solid condition of Si and α-Al particles was constant. Meanwhile, the eutectic mixture phase melted over time. The molten eutectic mixture was arranged during the holding time. A uniformly distributed presence of an almost globular shape of the primary α-Al phase is observed in the thixoformed microstructure. The optical micrographs in Figure 3a show the microstructure at the centre of the Al–7Si alloy billet. The rosette- and globular-like primary phase formed during cooling slope casting shows considerable spheroidization and coarsening due to the reheating process. Different from the mould cast sample, the microstructure of the thixoformed samples is free from porosity. Moreover, the solid particles are frequently observed to agglomerate in thixotropic alloys [32]. Figure 3a shows the agglomeration of some α-Al grains. This agglomeration is brought about by shearing and sintering effects that cause particle collision and solid bonding [33]. The eutectic phase of the thixoformed samples is finer than that of the cooling slope casting, and the rapid cooling of the samples from a semisolid state mainly produced this difference. The refinement of the microstructure is determined primarily by the solidification rate; it significantly affects the quality of the materials. A higher cooling rate produces a finer microstructure [34].

Figure 3b shows how the morphology of Si particles has been significantly modified from large individual flakes in the traditional mold cast alloy to a small acicular or skeleton network at increased magnification of the eutectic zone. The thixoformed samples have primary α-Al globules with an average size of 71 μm in diameter. The fine particles of acicular Si morphology have an average size of 2.51 ± 0.76 μm. The cooling rate in the bulk increases due to the pressure applied by the ram during thixoforming, promoting contact between the die wall and the melt. This condition may be attributed to the larger coefficient of heat transfer [35,36]. In addition, the short period of grain growth resulted in a more refined primary α-Al due to the cooling rate under pressure. The application of pressure produced a more refined eutectic Si with a rosette-like appearance, which is indicated by the red circle in Figure 3b, instead of the lamellar-like appearance [37]. The nucleation of eutectic Si produced very fine lamellar-shaped Si. This refinement can be obtained through a high local cooling rate.

### 3.4. Microstructure after the Heat Treatment

**Figure 4:**
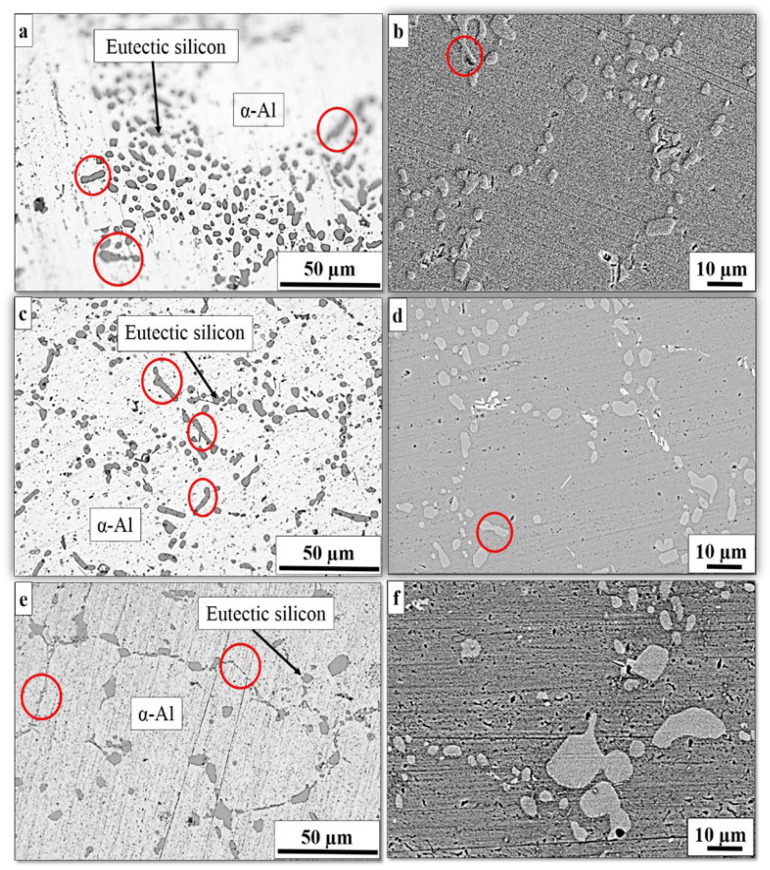
Optical and SEM micrographs of heat-treated eutectic Si particles in (**a**,**b**) as-cast, (**c**,**d**) cooling slope and (**e**,**f**) thixoformed Al–7Si alloy samples.

Figure 4 shows the SEM and optical micrographs of eutectic Si in the as-cast, cooling slope and thixoformed Al–Si alloy (300 series) after the T6 solution heat treatment. Compared with Figure 1, Figure 2 and Figure 3, transformation mostly occurred on a lamellar and relatively round shape of eutectic Si particles in the cooling slope and thixoformed, or the flak-like in as-cast into a spheroidized form. This transformation occurred after the application of the T6 solution heat treatment. A number of eutectic Si particles remained in a fine lamellar-like shape, as illustrated in Figure 4a–f. The result in this figure supports the findings presented by other researchers [27]. During the first stage of T6 heat treatment, the Mg_2_Si component and Si dissolve at a solutionizing temperature in the α–Al phase. The Si solid solubility value in the Al matrix is approximately 1.5 wt.%, whereas the solid solubility value of Mg_2_Si is approximately 1.4 wt. percent [38]. The T6 solution heat treatment in the Al–Si–Mg alloy was used to illustrate the occurrence of three phenomena: (i) precipitation of hardened Mg_2_Si particles during the second stage of T6 heat treatment, (ii) eutectic Si particle fragmentation and (iii) chemical homogenization. Furthermore, in thixoforming procedure, the size of the Si particles, which did not melt upon reheating, was maintained at a significant extent [39]. In addition, a number of large Si particles exhibited smooth edges, as illustrated in Figure 4e,f. The Si particle shape was influenced by (i) the different levels of pressure applied during the thixoforming process, (ii) the heat treatment used and (iii) the solidification rate. The minimisation and fragmentation of the lamellar Si plate-like into smaller pieces started during the solution treatment, followed by continuous spheroidization.

**Figure 5:**
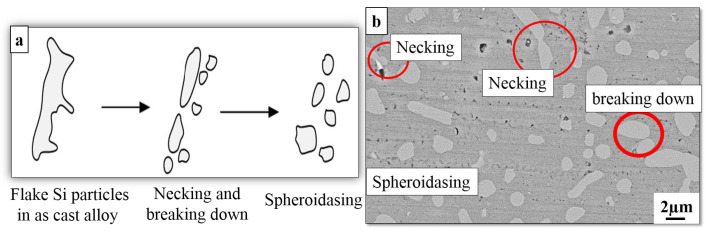
(**a**) Schematic characterization of three stages of spheroidization and coarsening of eutec-tic Si particles and (**b**) necking and break down of Si particles during T6 heat treatment.

Figure 5a displays the schematic of the coarsening and spheroidization process of Si particles. In the coarsening process, the development of larger particles occurred at the expense of the other small particles [40]. Consequently, the number of Si particles is reduced during the heat treatment process with a corresponding increase in the average particle size; meanwhile, the minimization and necking of Si particles during the T6 solution heat treatment are displayed in Figure 4b. Table 1 shows the quantitative metallography evaluation of microstructural attributes with and without the T6 treatment. It also presents the quantitative metallography evaluation of microstructural attributes with and without the T6 treatment. A decrease in diameter equivalent to Si particles occurred compared with that of the semisolid state and as-cast processes.

### 3.5. Effect of the Heat Treatment on the Intermetallic Compounds

**Figure 6:**
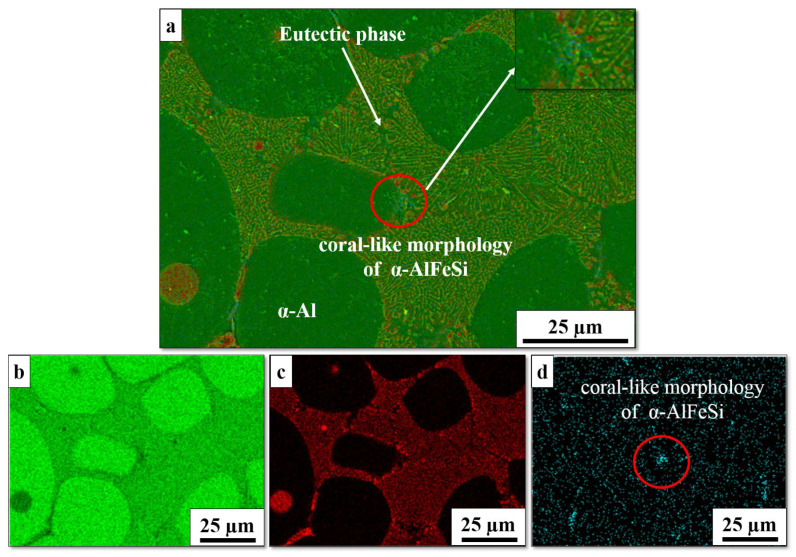
SEM-EDS elemental mapping of Al–7Si alloy: (**a**) SEM morphology of Fe-intermetallic; (**b**) Al; (**c**) Si; (**d**) Fe.

Heat treatment is carried out to achieve three benefits, homogeneity of the as-cast structure, dissolving of certain intermetallic particles, such as Mg_2_Si, and spheroidization of the eutectic Si morphology. However, a fourth benefit is obtained during heat treatment, namely, the transformation from the π-AlFeSiMg phase to the fine needle-like β-AlFeSi or α-AlFeSi phases, resulting in slight improvements in the mechanical properties after T6 heat treatment [21,41,42,43]. The transformation occurs in a midrange of Mg content (0.35–0.55 wt.%). Figure 6 shows the transformation, where π-AlFeSiMg partially transformed into α-AlFeSi, which has a coral-like morphology, due to the dissolution of Mg into the α-Al phase in the thixoformed sample. The finding is consistent with that of other researchers [21,41]. The Fe-intermetallic phase is affected by iron concentration and the cooling rate; low Fe concentration and a high cooling rate decreases the size of the Fe-phase [42]. A high cooling rate in the thixoformed sample leads to a refined Fe-intermetallic phase. This microstructural change is expected to considerably enhance the workability and processability of the alloy given that plate-like particles, such as β-AlFeSi, may lead to the initiations of local cracks and even generate surface defects [44].

### 3.6. Effect of Refinement Microstructure on the Hardness

**Figure 7:**
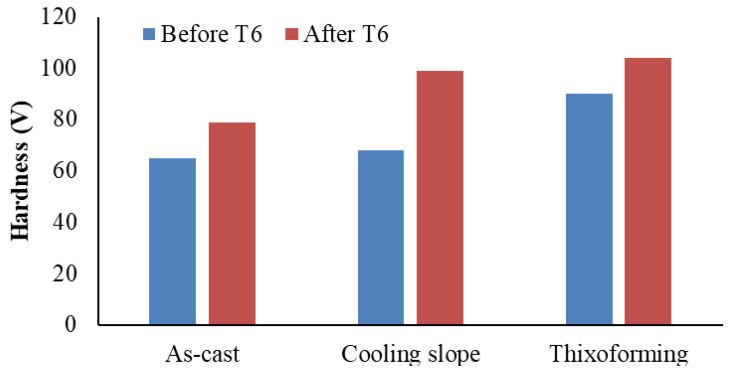
Microhardness of as-cast, cooling slope and thixoformed Al–7Si alloy before and after T6.

Figure 7 shows the average Vickers microhardness of the as-cast and semisolid Al-7Si alloy samples before and after heat treatment. The increase in hardness of the Al–7Si alloy after cooling slope and thixoformed processing is due to the microstructural transformation from coarse dendrite to globular α-Al altered Si particle size and morphology, as well as structural homogeneity. Alloy hardness increased from 65 HV to 86 and 90 HV, as shown in Table 2, after cooling slope and thixoformed processing, respectively. Shear force is able to break the dendrite arms of the α-Al phase, thereby refining the grain of the alloy. As a consequence, the microstructure in the cooling slope condition grows smaller and denser than that of the as-cast sample, and the microstructure of the cooling slope sample has the maximum microhardness [45]. The transformation of Si particles from a flake shape in as-cast to lamellar or acicular shapes in the cooling slope samples enhanced the microhardness of the Al–7Si alloy. The highest pressure imposed on the thixoformed sample increases the cooling rate, resulting in refined α-Al grains. Consequently, the Si morphology is transformed into a fine acicular shape. Furthermore, thixoforming eliminates porosity and shrinkage during the casting process, thereby increasing hardness. Evidently, spheroidization of eutectic Si after T6 heat treatment results in enhanced hardness of the samples. The potential for spheroidization of Si particles following T6 heat treatment and the precipitation of Mg_2_Si particles during the aging process boosts ultimate tensile strength and hardness [46,47].

### 3.7. Effect of Refinement Microstructure on the Corrosion

#### 3.7.1. Immersion Test Results Analysis

**Figure 8:**
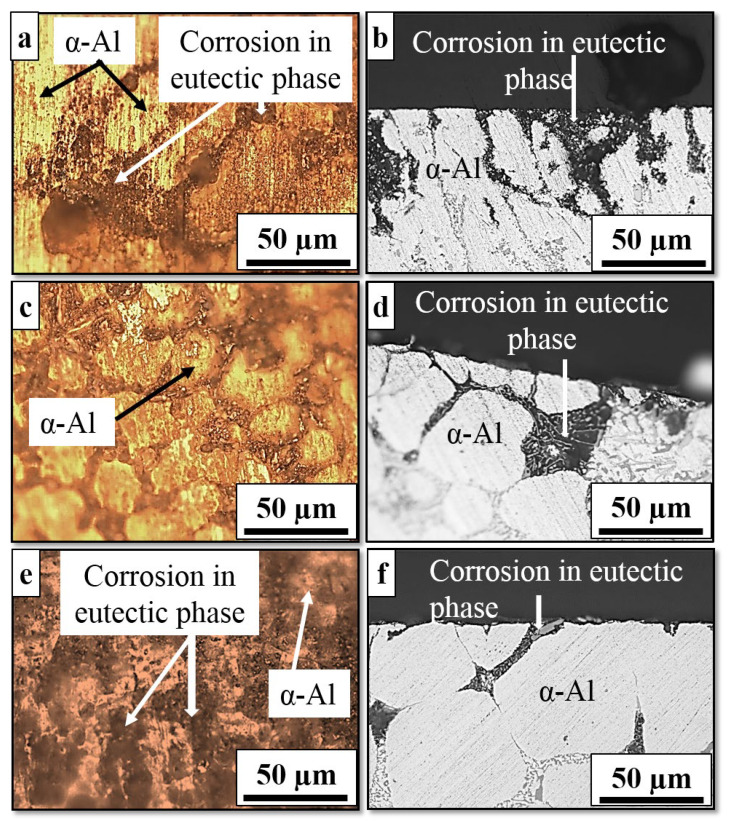
Optical micrographs of the surface morphology (top and side view) of (**a**,**b**) as-cast, (**c**,**d**) cooling slope and (**e**,**f**) thixoformed after 10 days of immersion in 3.5% NaCl solution.

The optical micrographs in Figure 8 show the top and side views of the surface morphology of as-cast, cooling slope and thixoformed samples after a 10-day immersion in 3.5% NaCl solution.

Selective corrosion attack is evident in the eutectic phase of the as-cast, cooling slope and thixoformed samples, whereas the primary α-Al phase remains unattacked. The presence of a relatively large eutectic phase, as shown in Figure 8b,d,f, promotes wider corrosion on the surface of the as-cast sample than the thixoformed and cooling slope samples. The cooling slope and thixoformed samples have a smaller area of localized corrosion compared to the as-cast sample. The initial corrosion in the as-cast, cooling slope and thixoformed Al–7Si alloys eventually spreads throughout the eutectic mixture [48]. Stable pits formed on the surface of the three alloys due to localized corrosion are a result of the galvanic corrosion action between the active phase (eutectic phase) and noble phase (Si and intermetallic compound particles) [49]. Larger localized corrosion pits are observed in the as-cast sample.

#### 3.7.2. Electrochemical Test Results Analysis

**Figure 9:**
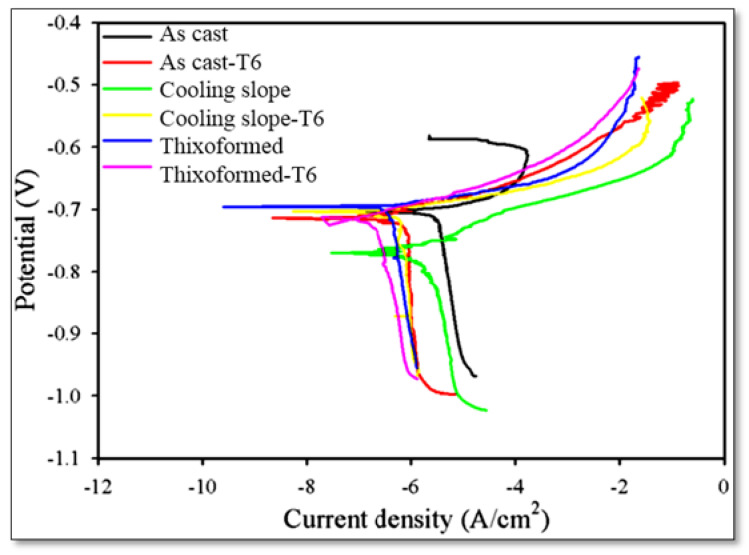
Polarization curves of Al–7%Si alloy before and after heat treatment in 3.5 wt.% NaCl.

The electrochemical behaviour of the Al–7Si alloy is evaluated by exposing the samples to a corrosive environment, simulating the seawater environment by using 3.5% NaCl electrolyte solution at ambient temperature. The Tafel extrapolation method is used in conjunction with the linear polarization approach to determine the corrosion resistance of as-cast, cooling slope and thixoforming samples. Figure 9 shows the polarization curves for as-cast, cooling slope-cast and thixoformed samples before and after heat treatment. The curves show that the estimated average corrosion potentials are almost identical, with only minor differences. Table 3 presents the corrosion rates of these samples. The results indicate that the reduced corrosion rate after semisolid processing can be attributed to the change in microstructure from a dendritic coarse structure to a fine globular one and the altered Si particle shape from a flaky to an acicular shape. The significantly refined Si particles and reduced eutectic phase area may be ascribed to the lower corrosion rate and improved polarization resistance, following T6 heat treatment processing of as-cast, cooling slope-cast and thixoformed samples. This condition could be due to the smaller cathodic to anodic area ratio [10,50]. The 0.0424 mmpy^−1^ corrosion rate of the as-cast alloy reduced to 0.0195 mmpy^−1^ after cooling slope and 0.0193 mmpy^−1^ after thixoformed processing. The corrosion rates following T6 heat treatment are 0.0160, 0.0079 and 0.00736 mmpy^−1^, due to the reduction in the area ratio of cathode to anode.

## 4. Conclusions

1. The primary dendritic α-Al phase morphology in the Al–7Si alloy as-cast sample was transformed into a fine globular shape in the cooling slope process using a cooling slope. However, in the thixoformed sample, due to heating, the primary α-Al phase morphed into a roughly spherical shape that was equally distributed and surrounded by the eutectic phase.
2. The coarse flake-shaped Si particles in as-cast sample were changed into lamellar-like shaped particles, measuring 3.2 ± 0.57 μm after cooling slope. The highest pressure imposed on the thixoformed sample increased the cooling rate, transforming the Si particles into fine acicular-shaped particles, measuring 2.51 ± 0.75 μm.
3. During the T6 heat treatment process of the Al–7Si alloy, the eutectic flaky-, lamellar- and acicular-like shaped eutectic Si particles were fragmented and spheroidized via a coarsening and spheroidization process measuring 2.32 ± 0.34 and 1.92 ± 0.87 μm, respectively.
4. The increase in hardness of the Al–7%Si alloy after cooling slope and thixoformed processing is due to the microstructural transformation from coarse dendrite to globular α-Al, altering Si particle size and morphology as well as structural homogeneity. The alloy hardness increased from 65 HV to 99 HV and 104 HV after the cooling slope and thixoformed process, respectively.
5. The presence of a relatively large eutectic phase promotes wider corrosion on the surface of the as-cast sample than the thixoformed and cooling slope samples. Different from the as-cast sample, the cooling slope and thixoformed samples have a smaller area of localized corrosion. The substantially refined Si particles and reduced eutectic phase area may be ascribed to the lower corrosion rate and improved polarisation resistance of as-cast, cooling slope and thixoformed samples following T6 heat treatment processing. This finding could be due to the smaller cathodic to anodic area ratio.

## Figures and Tables

**Table 1:** Average size of Si particles.

Samples	Si Size (µm)
As-cast	4.56 ± 1.18
As-cast—T6	2.76 ± 0.68
Cooling slope	3.2 ± 0.57
Cooling slope—T6	2.32 ± 0.34
Thixoformed	2.51 ± 0.76
Thixoformed—T6	1.92 ± 0.87

**Table 2:** Average of microhardness.

Samples	Microhardness (HV)
As-cast	65
As-cast—T6	79
Cooling slope	86
Cooling slope—T6	99
Thixoformed	90
Thixoformed—T6	104

**Table 3:** Average of corrosion rate (CR), current density (*i*corr.) and polarization resistance (R*p*).

Samples	Ecorr. (V)	*i*corr. (A/cm^2^)	R*p* (Ω·cm^2^)	CR (mm·y^−1^)
**As-cast**	−0.698	3.894 × 10^−6^	5.212 × 10^3^	0.04240
**As-cast—T6**	−0.710	8.432 × 10^−7^	2.035 × 10^4^	0.01600
**Cooling slope**	−0.769	1.790 × 10^−6^	9.690 × 10^3^	0.01950
**Cooling slope—T6**	−0.702	7.258 × 10^−7^	2.180 × 10^4^	0.00790
**Thixoformed**	−0.697	1.770 × 10^−6^	2.522 × 10^4^	0.0193
**Thixoformed—T6**	−0.742	6.587 × 10^−7^	3.779 × 10^4^	0.00740

## Data Availability

Not applicable.
